# Systemic Relapse in a Young Adult Patient with Primary CNS Diffuse Large B-Cell Lymphoma

**DOI:** 10.1155/2022/7139661

**Published:** 2022-06-21

**Authors:** Adam Khorasanchi, Zachary Benson, Misty Hall, Nelya Ebadirad, Mohammad H. Gharavi, Patrick Willard, Miranda Chimzar, John McKay, Gary Simmons, Victor Yazbeck

**Affiliations:** ^1^Department of Internal Medicine, Virginia Commonwealth University, Richmond, VA, USA; ^2^Department of Pathology, Virginia Commonwealth University, Richmond, VA, USA; ^3^Department of Radiology, Virginia Commonwealth University, Richmond, VA, USA; ^4^School of Medicine, Virginia Commonwealth University, Richmond, VA, USA; ^5^Massey Cancer Center, Virginia Commonwealth University, Richmond, VA, USA

## Abstract

Primary central nervous system diffuse large B-cell lymphoma (PCNS-DLBCL) is a rare form of non-Hodgkin's lymphoma, characterized by an aggressive disease course. While CNS relapse is common, systemic relapse is rare with no consensus on optimal treatment. This paper presents an unusual case of advanced PCNS-DLBCL with systemic relapse, including adrenal gland involvement. A review of the existing literature and a discussion on the management of systemic relapse in PCNS-DLBCL is also provided.

## 1. Introduction

Primary central nervous system lymphoma (PCNSL) is a rare and aggressive form of non-Hodgkin's lymphoma (NHL) confined to the brain, eyes, spinal cord, leptomeninges, or cranial nerves. [1] 90% to 95% are classified histologically as diffuse large B-cell lymphoma (PCNS-DLBCL) [2]. High-dose methotrexate (HD-MTX) based induction chemotherapy is the preferred first-line treatment. Despite high response rates to initial treatment, up to 60% of patients eventually relapse, with no standardized salvage regimen [3]. Patients with relapsed disease have an extremely poor prognosis with a median survival of 2 months without additional treatment [4].

While CNS relapse is common, systemic relapse is rare, occurring in 7–10% of patients and also represents a major therapeutic challenge [5]. Novel insights into the pathophysiology of PCNSL have identified the B-cell receptor (BCR) pathway as a key mechanism in the pathogenesis of PCNS-DLBCL. The use of targeted agents, such as ibrutinib and immunomodulatory drugs, has demonstrated promising clinical activity and offers additional therapeutic options in patients with relapsed/refractory disease [4].

This paper reports an unusual case of advanced PCNS-DLBCL with systemic relapse, including adrenal gland involvement. A review of the existing literature and a discussion on the management of systemic relapse in PCNS-DLBCL is also provided.

## 2. Case Report

**Figure 1:**
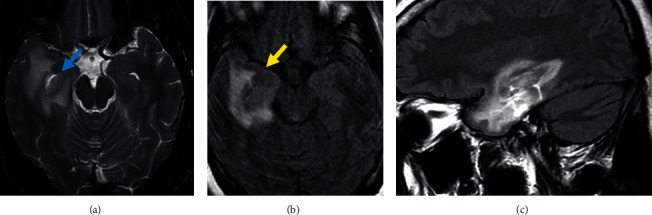
Axial T2-weighted (a), axial FLAIR (b), and sagittal FLAIR (c) views of MRI brain showing CNS relapse. Images show T2 isointense-hypointense signal in right inferior temporal lobe with peripheral edema causing expansion of the uncus (yellow arrow) without mass effect on temporal horn of right lateral ventricle (blue arrow).

**Figure 2:**
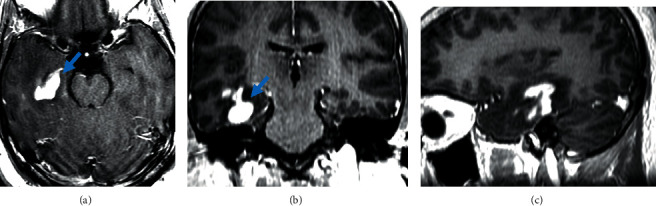
Contrast-enhanced axial (a), coronal (b), and sagittal (c) views of MRI brain showing CNS relapse. Images reveal homogenous enhancement (blue arrows) in periventricular tissues of right temporal lobe.

**Figure 3:**
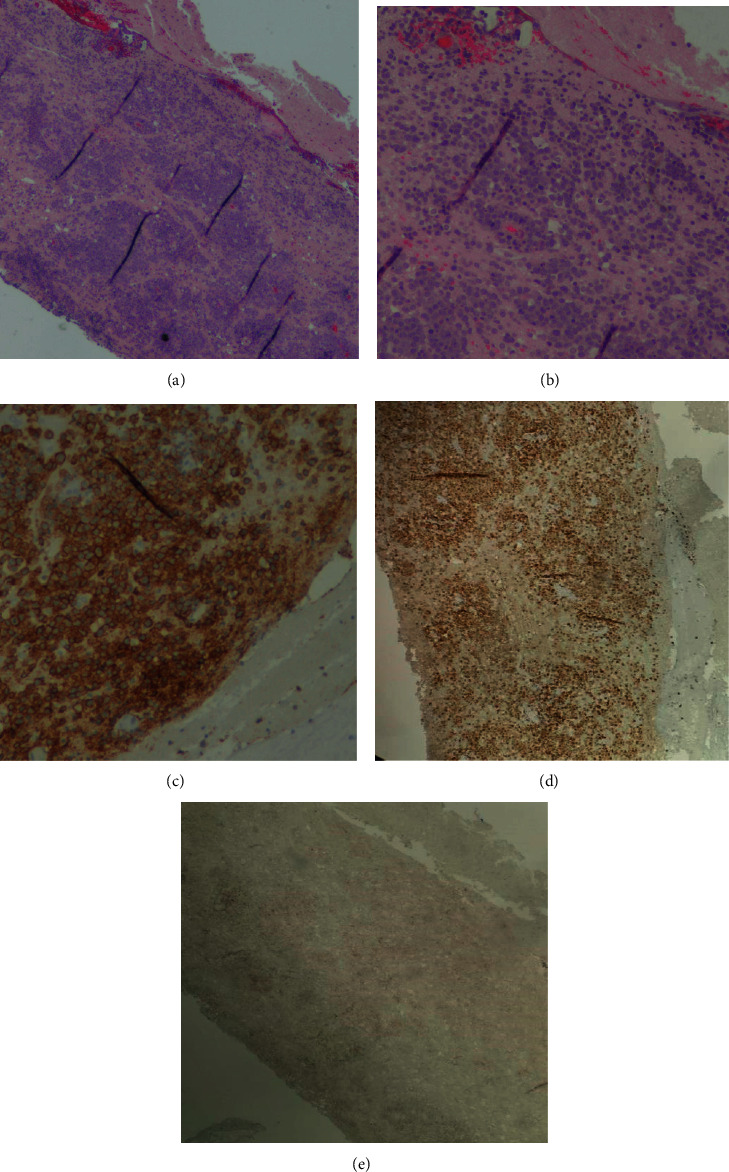
Parietal core brain biopsy of initial PCNSL mass. *H* and *E* stain showing neural tissue with infiltration of large round blue cells at 40x (a) and 100x (b). Positive CD20 stain highlighting the infiltration of large round blue cells at 100x (c). Positive MUM1 (d) and negative CD10 stains (e) supporting non-GCB subtype.

**Figure 4:**
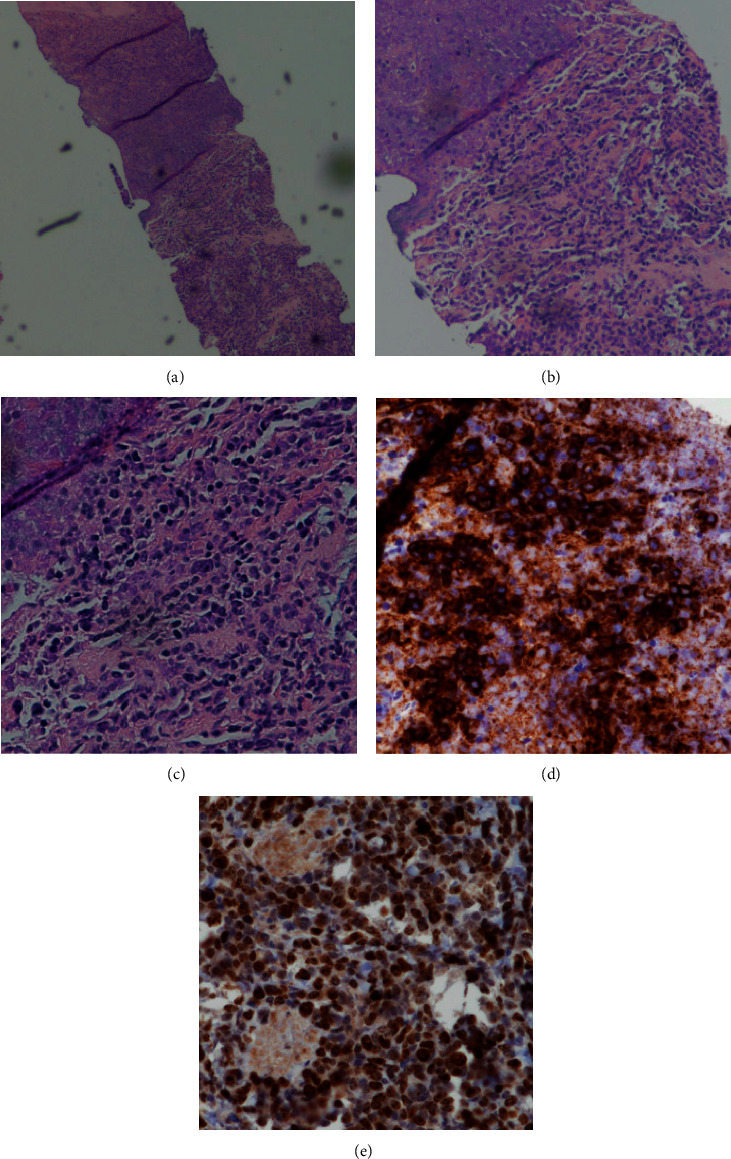
FNA and core biopsy of adrenal gland mass. *H* and *E* stain showing infiltration of large round blue cells and necrosis at 40x (a), 100x (b), 400x (c). CD20 stain highlight the infiltration of large round blue cells at 100x (d). Ki-67 stain showing a proliferation rate of >90% of the infiltration of large round blue cells at 100x (e).

**Figure 5:**
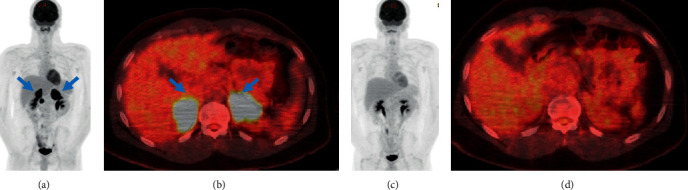
Pretreatment coronal maximum-intensity-projection (a) and axial fused FDG-PET (b) images show enlarged and hypermetabolic bilateral adrenal glands (blue arrows) with SUV max of 17.1 and 14.1 on the right and left respectively. Posttreatment coronal maximum-intensity-projection (c) and fused axial FDG-PET (d) images show resolution of previously seen hypermetabolic activity of bilateral adrenal glands two months following CAR-T.

A 23-year-old African-American male presented to our clinic following a diagnosis of relapsed CNS lymphoma (Figures 1 and 2). The patient's initial workup showed no evidence of systemic lymphoma. The patient had a history of Wolff–Parkinson White syndrome (WPW) and PCNS-DLBCL (nongerminal center B-cell-like (non-GCB) subtype, Figure 3), and had previously been treated with HD-MTX-based induction chemotherapy and an autologous stem cell transplant (ASCT). The patient maintained a complete response (CR) for 12 months prior to the diagnosis of CNS relapse. The patient was treated according to the radiation therapy oncology group (RTOG) 0227 protocol of preirradiation chemotherapy with HD-MTX, rituximab, and temozolomide for 6 cycles, followed by low-dose whole-brain radiation (WBRT) and postirradiation temozolomide. Posttreatment magnetic resonance imaging (MRI) showed no evidence of CNS disease. One year following therapy, the patient presented to the hospital with complaints of right upper quadrant abdominal pain and emesis. Imaging showed new bilateral adrenal masses. The biopsy demonstrated diffuse lymphoid infiltrate with extensive necrosis on hematoxylin and eosin (H&E) staining. Immunohistochemistry (IHC) detected PAX5+ and CD20+ cells (Figure 4), consistent with systemic relapse from his initial CNS lymphoma. Positron emission and computed tomography (PET-CT) showed increased tracer uptake of bilateral adrenal glands with a small mass inferior to the right adrenal gland (Figure 5). Given the non-GCB subtype, the patient was started on ibrutinib 560 mg daily as a bridge to chimeric antigen receptor T-cell therapy (CAR-T). The lymphodepleting regimen, fludarabine 30 mg/m^2^/d and cyclophosphamide 300 mg/m^2^/d, was administered for 3 days followed by an infusion of 3.6 × 10^8^ CAR-T cells/kg tisagenlecleucel (Kymriah). The patient's hospital course was complicated by supraventricular tachycardia associated with WPW and grade 1 cytokine release syndrome on day +6, requiring supportive treatment [6]. The patient achieved a complete metabolic response (CMR) on day +60 by PET-CT and MRI with a Deauville score of 3 (Figure 5). Unfortunately, on day +90 the patient was hospitalized with right hip pain and relapsed again with a right psoas mass biopsy proven DLBCL. The patient was treated with radiotherapy (total 36 Gy) to the psoas mass, but this was discontinued due to interval enlargement of the mass and the development of new confluent celiac lymphadenopathy. Next, the patient was started on salvage chemo-immunotherapy with rituximab-ifosfamide, carboplatin and etoposide (R-ICE), as a bridge to an allogeneic stem cell transplant. Following four cycles of R-ICE, the patient achieved near complete resolution of the psoas mass on PET-CT but developed worsening dizziness with a new right cerebellar mass on MRI. This was treated with radiotherapy (total 36 Gy) and one cycle of HD-MTX. Unfortunately, posttreatment MRI showed multiple new lesions in the hypothalamus, lateral ventricles, and enlargement of a known right subcortical parietal mass. The patient died from disease progression on day +310, nearly four years following the initial diagnosis.

## 3. Discussion

PCNS-DLBCL is characterized by an aggressive disease course and overall long-term survival rates that range from 20% to 40% [5]. Age, performance status, lactate dehydrogenase levels, and elevated cerebrospinal fluid protein are variables used in prognostic models [7]. Risk factors include immunodeficiency states, such as HIV infection. Mutations in the B-cell receptor and Toll-like signaling receptor pathways promote tumor development [7]. Histopathologic examination typically reveals perivascular cuffing of neoplastic cells with an almost constant expression of pan-B-cell markers (CD19, CD20, CD22, and CD79a) on IHC [1]. B-cell lymphoma 6 (BCL6) positivity is seen in 60% of tumors and has been associated with a worse prognosis. Interferon regulatory factor 4/multiple myeloma oncogene 1 and BCL2 are positive in 95% of tumors. Increased MYC expression is also common [7]. Two distinct molecular subtypes of PCNS-DLBCL exist: GCB and non-GCB, with the non-GCB subtype (>85%) being more common and associated with poor overall survival (OS) [8, 9].

Commonly reported symptoms in patients with PCNS-DLBCL include focal neurologic deficits, neuropsychiatric symptoms, and signs of elevated intracranial pressure such as headache, nausea, and vomiting [3]. The classic “B” symptoms are rarely seen [4]. Lesions on contrast-enhanced MRI are often periventricular and involve the deep white matter, basal ganglia, or corpus callosum [3]. A definitive diagnosis of PCNS-DLBCL requires a brain biopsy. An ophthalmic exam, lumbar puncture, whole body PET/CT, and bone marrow examination are recommended to rule out systemic disease [4, 7].

Management of frontline PCNS-DLBCL involves a multimodal approach including chemotherapy, stem cell transplantation, and/or radiotherapy [10]. The role of surgery is limited to diagnostic purposes due to the diffuse and infiltrative disease nature [3]. While PCNS-DLBCL is extremely sensitive to radiotherapy, this modality alone is no longer recommended for initial treatment, given the lack of durable responses and increased risk of neurotoxicity [10]. However, WBRT remains a reasonable alternative in the frontline setting for: (1) patients who are not candidates for HD-MTX-based induction; (2) in the consolidation setting; or (3) as salvage therapy in patients who have not responded adequately to induction chemotherapy [10].

The first step in the management of PCNS-DLBCL is induction chemotherapy with the goal of achieving a complete radiographic response [11]. A HD-MTX-based regimen is the preferred first-line induction therapy for newly diagnosed cases. Following induction therapy, most patients require consolidation treatment to eliminate residual disease and prolong overall survival (OS) [11]. If CR is achieved, consolidation options include: (1) high-dose chemotherapy with ASCT (in younger patients and patients with adequate organ function), (2) high-dose cytarabine with etoposide, (3) low-dose WBRT, or (4) continuous monthly HD-MTX after induction therapy. If residual disease is present, WBRT is recommended [12]. Ongoing trials that randomly assign patients to different consolidation treatments will hopefully shed more light on the optimal consolidation regimen. In addition, age and response to induction therapy should be used to guide the choice of consolidation [2].

Despite such therapy, nearly 50% of patients relapse with a median time to relapse of 10–18 months [4]. There is no consensus on optimal treatment following relapse. The choice of salvage treatment depends on age, previous treatment and response, performance status, relapse site (CNS vs. systemic), and comorbidities. Salvage treatment for CNS relapse may include chemotherapy, radiotherapy, stem cell transplantation, or immunotherapy [10]. For MTX-sensitive disease, retreatment with HD-MTX-based regimens should be considered, especially if relapse occurred more than one year after a HD-MTX-based regimen [3]. In a retrospective analysis, rechallenge with HD-MTX led to a significant overall response rate (ORR) of 85–91%, with a median OS of 41–62 months [13, 14]. High-dose cytarabine, either alone or used in combination, has been associated with CR rates of 40–70% [15–19]. Modest activity has also been observed with single-agent bendamustine in a study of 12 patients with relapsed PCNSL with half of the patients responding [20]. Our patient's initial CNS relapse was treated as per the RTOG 0227 protocol. Results from this study for previously untreated PCNSL demonstrated a two year OS rate of 80.8% and a 51% CR with minimal rates of neurotoxicity [21].

While most patients experience CNS relapse, systemic relapse is rarely observed, as seen in our patient with adrenal and psoas involvement [22]. Additional sites of systemic relapse reported in the literature include musculoskeletal, testicular, kidney, and liver involvement [23]. Interestingly, there are few published cases of adrenal relapse. Ma et al. described a patient found to have bilateral adrenal involvement of their PCNS-DLBCL following MTX-based chemotherapy and WBRT, who was started on rituximab-hyper CVAD and then transitioned to R–CHOP [5]. In patients found to have bilateral adrenal involvement, primary adrenal lymphoma (PAL) with secondary CNS involvement should also be considered in the differential [5]. In fact, adrenal involvement has been found to be highly associated with CNS relapse risk and is one of the criteria used in the CNS international prognostic index for DLBCL [24]. Our patient's adrenal biopsy results showed non-GCB subtype which was consistent with his prior PCNSL, and therefore confirmed secondary rather than primary adrenal involvement.

Management of systemic relapse in PCNS-DLBCL is challenging given its rarity and lack of effective treatment options. Patients with systemic relapse in PCNSL-DLBCL are treated similarly to those with relapsed systemic DLBCL [23]. Myeloablative chemotherapy and ASCT are considered important components in first-line and later therapy [25]. Allogeneic stem cell transplant may also be curative and remains an option for patients who relapse following ASCT [25]. An emerging new treatment option for systemic relapse is CAR-T-cell therapy. Studies of anti-CD19 CAR-T cells in relapsed systemic DLBCL have shown up to 50% CR and durable remission [26–28]. Due to safety concerns with immune effector cell associated neurotoxicity and unclear efficacy in patients with a history of advanced CNS disease, this cohort of patients has largely been excluded from CAR-T clinical trials [29]. However, subsequent work has demonstrated the safe and efficacious use of CAR-T in patients with CNS malignancies with appropriate management [30]. In an ongoing single center phase 1 trial, investigating the use of CAR-T in seven patients with a history of primary or secondary CNS lymphoma (SCNSL), an ORR of 57% was observed with no cases of grade ≥3 neurotoxicity [31]. Another study described 8 SCNSL patients who received CAR-T, none of whom experienced > grade 1 neurotoxicity. Two patients were noted to have CR at day +90 and day +180 respectively, while two patients died within 30 days secondary to disease progression [30]. Several ongoing clinical trials [32, 33] are exploring the use of CAR-T in PCNSL (Table 1).

Despite how promising CAR-T appears to be, 21–35% of patients still experience relapse, as seen with our patient [29]. Unfortunately, in those with multiple disease relapses, treatment outcomes remain poor [34]. Novel targeted therapies, which have demonstrated efficacy both within the CNS and systemically for relapsed-refractory (R/R) PCNSL, should be considered. These agents have been investigated in prospective clinical trials [29, 35–56], and are summarized below (Table 2). Single-agent temsirolimus, an mTOR inhibitor, demonstrated an ORR of 54% in a study of 37 patients with refractory PCNSL [44]. Ibrutinib, a Bruton's tyrosine kinase inhibitor, demonstrated excellent single-agent activity with an ORR of 77% in 13 PCNSL patients [45]. Additionally, ibrutinib was added to HD-MTX and rituximab in 15 patients with PCNSL and SCNSL. In PCNSL, 8 patients responded, including 6 patients achieving CR [46]. However, the durability of response to this regimen is unknown since responding patients were allowed treatment with ASCT consolidation [57]. Immunomodulatory drugs (IMiDs), which inhibit NF-K*β* activity, include lenalidomide and pomalidomide [57]. In a multicenter phase 2 study, 50 patients with relapsed PCNSL received lenalidomide and rituximab for eight cycles, followed by maintenance lenalidomide for responders. The observed ORR was 32%, including 13 achieving CR [49]. Pomalidomide and its combination with dexamethasone demonstrated significantly improved survival compared with IMiDs alone [50]. Finally, the use of immune checkpoint inhibitors (ICI) may represent another promising treatment approach in PCNSL [58]. In an immunocompetent preclinical model, anti-PD1 monoclonal antibodies had significant therapeutic activity against CNS lymphoma [59]. Moreover, Nayak et al. reported long-term responses in a small retrospective study of 4 patients with PCNSL [60].

## 4. Conclusion

This paper describes a rarely reported case of a 23-year-old man with advanced PCNS-DLBCL and systemic relapse, including adrenal gland involvement. Despite achieving near complete resolution of adrenal disease following CAR-T, the patient experienced multiple disease relapses and ultimately died from disease progression. Typically associated with a poor prognosis, this case highlights the challenges associated with management of systemic relapse in PCNS-DLBCL, and the necessity of well-designed clinical trials testing novel treatment options for this rare and often deadly disease.

## Figures and Tables

**Table 1:** Current CAR-T clinical trials in R/R PCNSL.

Sponsor	Study chair	Study design	Population	Conditions	Interventions	NCT
University College London	Claire Roddie	Phase I	Adults (>16 years)	(i) R/R PCNSL	Anti-CD19 CAR-T cells	NCT04443829
Massachusetts General Hospital	Matthew J. Frigault	Phase I	Adults (>18 years)	(i) R/R PCNSL	Tisagenlecleucel (anti-CD19 CAR-T cells)	NCT04134117
Dana-Farber Cancer Institute	Caron A. Jacobson	Phase I	Adults (>18 years)	(i) R/R CNSL (ii) systemic lymphoma with concurrent CNSL	Axicabtagene ciloleucel (anti-CD19 CAR-T cells)	NCT04608487
Memorial Sloan Kettering Cancer Center	Jae Park	Phase I	Adults (>18 years)	(i) R/R CNSL (ii) systemic lymphoma with concurrent CNSL	Anti-CD19 19 (T2) 28z1XX CAR-T cells	NCT04464200
Celgene	Claudia Schuster-bauer	Phase II	Adults (>18 years)	(i) R/R CNSL (ii) systemic lymphoma with concurrent CNSL	Lisocabtagene maraleucel (anti-CAR-T cells)	NCT03484702
Zhejiang University	He Huang	Phase I	(i) Children (>3 years) (ii) Adults (18–75 years)	(i) ALL with CNS involvement (ii) NHL with CNS involvement	Anti-CD19 CAR-T cells	NCT04532203
UNC Lineberger Comprehensive Cancer Center	Natalie Grover	Phase I	Adults (>18 years)	(i) R/R BCL (ii) R/R PCNSL (iii) R/R CLL/SLL	iC9-anti-CD19 CAR-T cells	NCT03696784
Shenzhen Genoimmune Medical Institute	Lung-Ji Chang	Phase I/II	(i) Children (>6 months) (ii) Adults (18–75 years)	(i) R/R BCL	4SCAR19 and 4scar20/22/70/PSMA/13/79b/GD2	NCT04429438

R/R: relapsed-refractory; PCNSL, primary CNS lymphoma; CAR-chimeric antigen receptor; NCT, national clinical trial identifier; ALL, acute lymphoblastic leukemia; NHL, non-Hodgkin lymphoma; BCL: B-cell lymphoma; CLL/SLL, chronic lymphocytic leukemia/small lymphocytic lymphoma; iC9: inducible caspase 9.

**Table 2:** Agents with activity in R/R PCNSL.

Author	Agents	Study type	^#^ of patients	ORR (PR + CR)	Median PFS, mo	Median OS, mo
Fischer et al. [35]	Topotecan	Prospective	27	9/27 (33%)	2	8.4
Voloschin et al. [36]	Topotecan	Prospective	15	6/15 (40%)	2 (60 d)	32.7
Reni et al. [37]	Temozolomide	Prospective	36	11/36 (31%)	2.8	3.9
Soussain et al. [38]	CYVE + SCT	Prospective	43	20/40 (50%)	11.6	18.3
Batchelor et al. [39]	Ritux	Prospective	11	4/11 (36%)	1.9 (57 d)	20.9
Raizer et al. [40]	Pemetrexed	Prospective	11	6/11 (55%)	5.7	10.1
Dietrich et al. [41]	Pemetrexed	Prospective	14	8/14 (57%)	4.2	44.5+
Rubenstein et al. [42]	IT Ritux + IT MTX	Prospective	14 (6 PCNSL)	6/14 (43%)	1.2	NR
Nayak et al. [43]	Ritux + temozolomide+pred	Prospective	16	5/14 (36%)	1.6 (7 wk)	NR
Korfel et al. [44]	Temsirolimus	Prospective	37	20/37 (54%)	2.1	3.7
Grommes et al. [45]	Ibrutinib	Prospective	20 (13 PCNSL)	10/13 (77%)	4.6	15 (PCNSL)
Grommes et al. [46]	Ibrutinib + HD-MTX + Ritux	Prospective	15	12/14 (80%)	9.2	NR
Roschewski et al. [47]	TEDDi-R : ibrutinib + anthracycline	Prospective	18	9/13 (69%)	15.2	NR
Rubenstein et al. [48]	Lenalidomide	Prospective	14	9/14 (64%)	6	NR
Ghesquieres et al. [49]	Revri : Lenalidomide + Ritux	Prospective	50	16/50 (32%)	7.5	14.7
Tun et al. [50]	Pomalidomide + dexamethasone	Prospective	29	12/25 (48%)	5.3	NR
Fox et al. [51]	Tier : Thiotepa + ifos + eto + Ritux	Prospective	27	14/27 (52%)	3	5
Kasenda et al. [52]	HDAra-C + Thiotepa + Ritux + SCT	Prospective	39	28/39 (72%)	12.4	NR
Gavrilenko et al. [53]	Nivolumab	Prospective	9 (8 PCNSL)	7/9 (78%)	12	12
Siddiqi et al. [54]	CD19 CAR-T-cell therapy	Prospective	7 (3 PCNSL)	4/7 (57%)	NR	NR
Li et al. [55]	CD19/CD22 CAR-T-cell therapy	Prospective	5 (1 PCNSL)	5/5 (100%)	3	NR
Tu et al. [29]	CD19/CD70 CAR-T-cell therapy	Case study	1	1/1 (100%)	>17	>17
Siddiqi et al. [56]	CD19 CAR-T-cell therapy	Case series	5	3/5 (60%)	NR	NR

ORR, overall response rate; CR, complete response; PR, partial response; PFS, progression free survival; OS, overall survival; NR, not reported; IT, intrathecal; pred, prednisone; HDAra-C, high-dose cytarabine; CYVE, cytarabine + etoposide; SCT, stem cell transplant; HD-MTX, high-dose methotrexate; Ritux, rituximab; ifos, ifosfamide; eto, etoposide; CAR, chimeric antigen receptor.
